# Co-prescribing of antidepressants and opioids for non-cancer pain in England, 2010–2019: a descriptive study using CPRD primary care electronic health records

**DOI:** 10.1186/s12875-025-02956-1

**Published:** 2025-08-16

**Authors:** Jake Butler, Rebecca M. Joseph, Carol Coupland, Roger David Knaggs, Anthony J. Avery, Richard Morriss, Debbie Butler, Louisa Gerrard, Dave Waldram, Ruth H. Jack

**Affiliations:** 1 Centre for Academic Primary Care, Lifespan and Population Health, School of Medicine, University of Nottingham, Nottingham, UK; 2 School of Pharmacy, University of Nottingham, Nottingham, UK; 3 NIHR Greater Manchester Patient Safety Collaboration, University of Manchester, Manchester, UK; 4 National Institute of Health and Care Research Nottingham Biomedical Research Centre, Nottingham University Hospitals NHS Trust, Nottingham, UK; 5 National Institute for Health and Care Research MindTech MedTech Co-operative, Institute of Mental Health, School of Medicine, University of Nottingham, Nottingham, UK; 6 Mental Health and Clinical Neurosciences, School of Medicine, University of Nottingham, Nottingham, UK

**Keywords:** Antidepressants, Opioids, Co-prescribing, Primary care

## Abstract

**Background:**

There is a complex relationship between pain and mood disorders, and interactions between opioids and antidepressants can affect the effectiveness and adverse effects of these medicines when taken together. However, little is known about the scale of co-prescription for these medicines.

**Methods:**

We used routinely collected primary care data from the Clinical Practice Research Datalink to describe the extent of opioid and antidepressant co-prescribing in over 4.3 million adults in England. Linked data included deprivation information and hospital episode statistics admitted patient care data to improve completeness of ethnicity information. We identified all primary care prescriptions of opioids and antidepressants between 2010 and 2019 and counted if an opioid and antidepressant prescription overlapped, and if so, for how long. People were censored at the first date of a record of cancer, terminal illness, heart failure or opioid misuse.

**Results:**

There were 4,355,694 people included in the study population. Of these, 304,029 (7.0%) had an opioid and antidepressant co-prescribed at least once during the study period. The prevalence of co-prescribing increased from 35.8 per 1000 person-years in 2010 to 44.1 in 2015 and then decreased to 39.2 in 2019. Co-prescribing rates were higher in females, older age groups, people living in more deprived areas and the White ethnic group. The overall median length of the opioid and antidepressant co-prescriptions was 29 days (interquartile range: 17 to 51 days). The most commonly co-prescribed medicines were codeine and amitriptyline, co-prescribed 235,017 times to 87,274 people. The second most commonly co-prescribed combination was codeine and citalopram, co-prescribed 55,792 times to 158,812 people. Combinations of opioids and antidepressants both metabolised by CYP2D6 were also common.

**Conclusions:**

There is a substantial group of people co-prescribed opioids and antidepressants in England, including combinations that may be less effective. This information will be useful to help GPs, dispensing professionals, policymakers and others understand how many people in the UK may be at risk of harm from using both types of medicines at the same time, and which groups are particularly affected. Future research should determine whether there are higher risks of adverse events in these co-prescribed groups.

**Supplementary Information:**

The online version contains supplementary material available at 10.1186/s12875-025-02956-1.

## Introduction

Antidepressant and opioid prescribing both present a significant public health concern, particularly when these medicines are taken at the same time [1]. This overlapping prescribing of medicines is referred to as co-prescribing. There is a complex relationship between pain and mood disorders, and little is known about how often patients are co-prescribed medicines for these conditions. Depression not only increases the likelihood of receiving opioid prescriptions but decreases opioid effectiveness [2, 3]. Pain also reduces responsiveness to antidepressants [4, 5] leading to complex prescribing patterns that could negatively affect patients. There are risks of adverse outcomes, such as serotonin syndrome, if opioids and antidepressants are taken in combination [6], with the United States Food and Drug Administration (US FDA) publishing warnings about co-prescribing in 2016, specifically around serotonin syndrome [7]. The combination of antidepressants that inhibit CYP450 2D6 (notably fluoxetine, paroxetine and to a lesser extent, duloxetine) and opioids (including codeine and tramadol) that require metabolism by CYP2D6 will be less effective and can potentially lead to adverse outcomes such as increased emergency hospital visits [8]. Another important risk of co-prescribing antidepressants and opioids is the risk of respiratory depression [9]. While risks are present with the co-prescribing of opioids and all psychotropic medicines [10, 11], opioids and antidepressants are some of the most commonly prescribed medicines in primary care. As such, there is interest as to how frequently people are co-prescribed these two classes of medicines and whether particular population groups are more likely to be co-prescribed.

Antidepressants are widely prescribed in England: in 2018, 70.9 million antidepressant prescriptions were dispensed, with a doubling of prescription rates over the previous decade [12]. In 2021, the National Institute for Health and Care Excellence (NICE) recommended the use of antidepressants as a first-line drug treatment for chronic primary pain for people over the age of 18 years, and recommended not initiating opioids in people aged over 16 years, in the UK [13]. It is possible that these new recommendations could further inflate antidepressant prescribing.

Opioids are also commonly prescribed and their use followed a similar increasing trend. A Public Health England report found 5.6 million people (12.8% of the population) were prescribed opioids (excluding those for the treatment of cancer pain) in 2017/18 [14]. A UK retrospective population-based study including 2 million new users of opioid medicines for non-cancer pain from 2006 to 2017 found opioid prescribing increased in this pre-Covid-19 pandemic period, with a 5-fold increase of codeine prescribing [15]. This study also found 25% of those prescribed moderate to high doses were still prescribed opioids after one year, while nearly 30% of those prescribed opioids had a documented history of depression and/or self-harm. A quarter were currently taking psychotropic medicines, mostly antidepressants. While more recent data shows the number of people in England being dispensed an opioid in primary care decreased between 2016/17 and 2020/21, the number total number of items dispensed remained stable at around 39 million a year in this period [16].

The extent of opioid and antidepressant co-prescribing in the general population in England over time has not been described, to our knowledge. Owing to the new NICE recommendation on antidepressants and the documented increases in opioid and antidepressant prescribing, co-prescribing may become more common. As such, this study aimed to describe the extent of co-prescribing of opioids for non-cancer pain and antidepressants within primary care in England, highlighting known high-risk combinations. We examined how opioid and antidepressant co-prescribing has changed over time, whether this varied by demographic factors, and whether people co-prescribed these medicines had a prior record of antidepressant indications.

## Methods

### Data source

Data came from the UK’s Clinical Practice Research Datalink (CPRD GOLD January 2023 (Version 2023.01.001) 10.48329/q5ta-3q58), a database of anonymised routinely collected primary care electronic health records. CPRD patients are representative of the UK population in terms of age, sex and ethnicity [17]. The data include demographic characteristics, symptoms and diagnoses, primary care issue prescriptions, test results, and other clinically important events. Linked data provided included deprivation information and hospital episode statistics (HES) admitted patient care data [18] from NHS England to improve completeness of ethnicity information.

### Study population

Our study population included all eligible males and females in CPRD GOLD aged 18–100 years in England between 1 st January 2010 and 31 st December 2019. Each patient’s study entry date was defined as the latest of the following: 1 year after registration with a practice, 1 year after the practice up-to-standard date (an indicator of data quality), 1 st January of the year they turned 18 years old or 1st January 2010. People were followed up until the earliest date of patient death, leaving the practice, practice last data collection, 1st January in the year they turned 101 years old or 31 st December 2019. Additionally, to try and focus on opioid prescriptions for non-cancer pain, we identified records of cancer, terminal illness, or opioid misuse disorder. We also excluded patients with recorded heart failure, as opioids are commonly used to manage breathlessness. Patients were excluded if they had any of these recorded before their study entry date, and were censored on the earliest of these dates if recorded during their follow-up.

### Outcome

The study outcome of co-prescribing was defined as having opioid and antidepressant prescriptions which overlapped by one or more days. We defined a list of opioids and antidepressants using the British National Formulary [19] which were used as search terms when compiling our drug lists (Additional file 1). These lists were verified by clinician and pharmacist members of the research team (RDK, RM, AA). The duration of prescriptions was estimated using an algorithm as detailed in Joseph et al. [20], adapted from an earlier published algorithm by Pye et al. [21], which uses information on quantity prescribed and daily dose to approximate prescription end dates given the known prescription issue date. In addition to defining the occurrence of overlapping opioid and antidepressant prescriptions, we calculated the number of days of overlap and determined which specific medicines were co-prescribed. We identified the most commonly co-prescribed combinations of opioids and antidepressants. Specific drug combinations identified as having an increased risk of a severe adverse event using Stockley’s interaction checker [22] were defined as being contraindicated (Additional file 1).

### Other variables

We examined co-prescribing by several patient characteristics. These were: sex (male and female), age in years (categorised into the following groups: 18–34, 35–44, 45–54, 55–64, 65–74, 75–84, 85–100 years), deprivation (Townsend deprivation quintile [23] at person-level, or if this was not available, practice-level), and ethnicity (Asian/Asian British, Black/Black British, Mixed, Other, White and not known). When ethnicity was not recorded in CPRD, this was supplemented using HES ethnicity information if available, and recorded as not known otherwise. Antidepressant indications (depression, anxiety/phobias and neuropathic pain) were defined using Read codes (Additional file 1).

### Analysis

We calculated prevalence and incidence rates of opioid and antidepressant co-prescribing per 1000 person years. Incidence rates were calculated for the first co-prescribing event during the study period and excluded patients with previous co-prescribing events. We calculated rates overall, by year, and by patient characteristics across the study period. We examined length of co-prescribing in days, reporting the median and interquartile range (IQR). Contraindicated combinations were reported using frequencies. For people with an incident co-prescription, we summarised the proportion with an indication for antidepressants recorded on or before the date of co-prescription. We also determined which medicine (opioid or antidepressant) was prescribed first. To distinguish between short- and long-term opioid prescribing, we determined whether the opioid had been prescribed for at least 12 months on the first date of co-prescription, if the opioid was prescribed first.

### Sensitivity analysis

As we found a large reduction in practices contributing to CPRD GOLD across the study period, we carried out a sensitivity analysis restricting our incidence and prevalence calculations to patients from practices contributing for the entire study period. We also limited the analyses to only include prescriptions that overlapped for at least 14, 28 and 365 days.

Stata 18 was used for data cleaning, analyses and creating incidence and prevalence rate figures. UpSet plots to display drug combinations were created in R using ComplexUpset [24, 25]. This study is reported using the REporting of studies Conducted using Observational Routinely collected health Data (RECORD) Statement (see Additional file 2, RECORD statement).

### PPIE (Patient and Public Involvement and Engagement)

The study team includes three public contributors (DB, LG, DW) who helped provide insights on our study objectives, design, methods, and interpretation, and are co-authors on this paper.

## Results

**Fig. 1:**
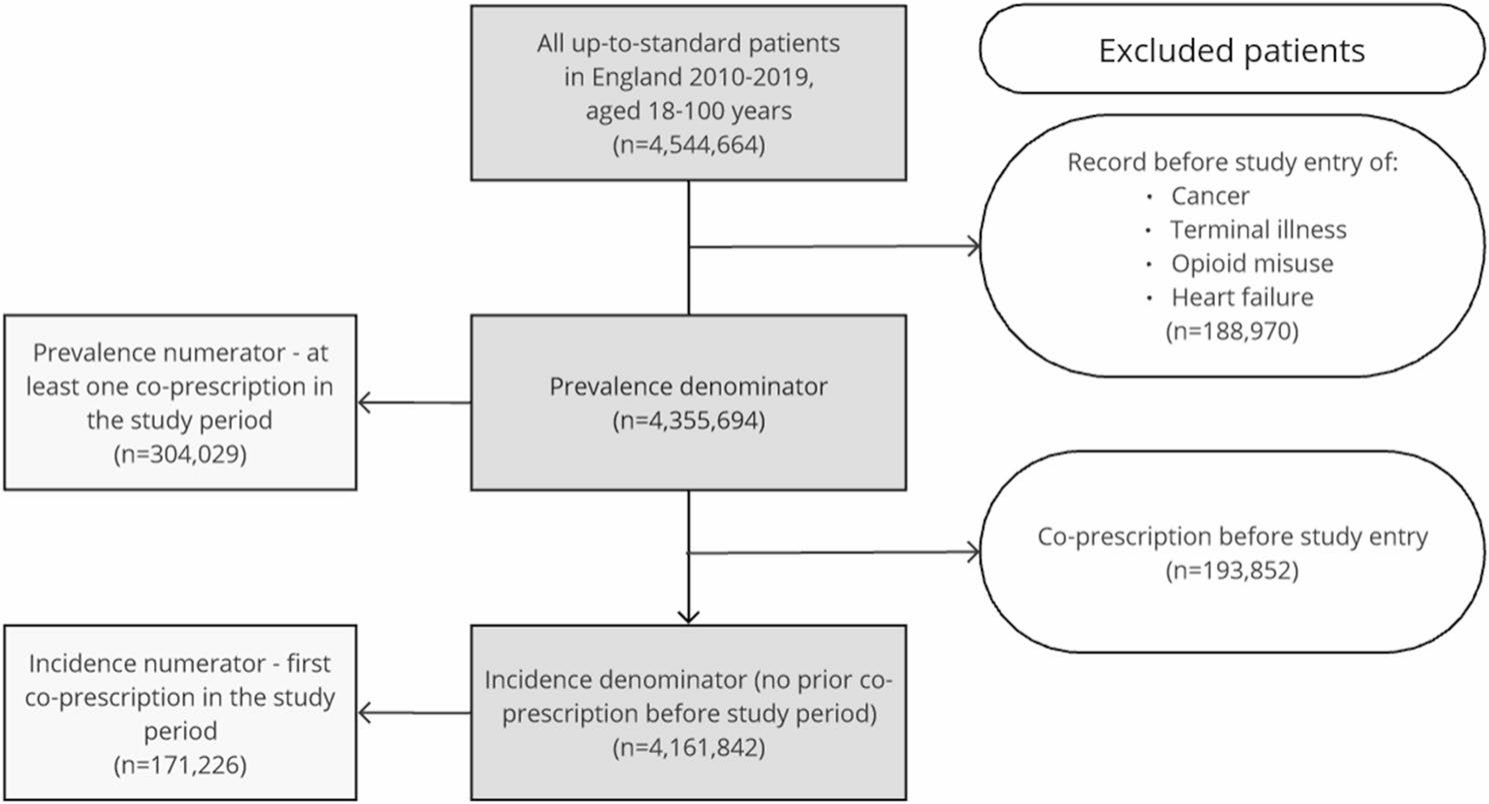
Flowchart of selection of study population

A flow chart detailing the selection of the study population is shown in Fig. 1. The dataset included 4,544,644 adults with up-to-standard records between 2010 and 2019. 188,970 patients were removed from this population owing to a record of an exclusion criteria prior to study entry, leaving a study population of 4,355,694 people and a follow-up period of over 17.5 million person-years (around 4 person-years on average). Of these, 304,029 (7.0%) had co-prescriptions during their follow-up. A further 193,852 people were excluded from incidence analyses due to having a co-prescription prior to the study period, and 171,226 (4.1%) of the remaining study population had incident co-prescriptions during follow-up. Ethnicity information was available in CPRD GOLD for 2,575,798 (59.1%) people in the study population. HES records supplemented ethnicity records for a further 952,739 (21.9%) patients, giving a recorded ethnicity for 81.0%. Details of characteristics of the study population overall and by whether they were co-prescribed opioids and antidepressants are shown in Table 1. The proportion of females was higher in the co-prescribed group compared with the remaining study population (68.1% v 49.6%). People who were co-prescribed opioids and antidepressants were also more likely to be older, White and to live in more deprived areas. These patterns were similar when restricting to people who had co-prescriptions lasting at least 90 or 365 days (Additional file 3, Table S3.1).

**Table 1:** Characteristics of prevalence cohort by co-prescription status, aged 18–100 years, England 2010–2019

	Co-prescribed an opioid and antidepressant					
	No		Yes		Total	
Overall	4,051,665	(100.0%)	304,029	(100.0%)	4,355,694	(100.0%)
Sex						
Male	2,042,095	(50.4%)	97,119	(31.9%)	2,139,214	(49.1%)
Female	2,009,570	(49.6%)	206,910	(68.1%)	2,216,480	(50.9%)
Age category at study entry (years)						
18–34	1,545,141	(38.1%)	44,816	(14.7%)	1,589,957	(36.5%)
35–44	753,496	(18.6%)	52,983	(17.4%)	806,479	(18.5%)
45–54	642,183	(15.8%)	63,043	(20.7%)	705,226	(16.2%)
55–64	490,872	(12.1%)	57,285	(18.8%)	548,157	(12.6%)
65–74	329,548	(8.1%)	44,253	(14.6%)	373,801	(8.6%)
75–84	196,786	(4.9%)	30,161	(9.9%)	226,947	(5.2%)
85–100	93,639	(2.3%)	11,488	(3.8%)	105,127	(2.4%)
Region						
North East	70,952	(1.8%)	6895	(2.3%)	77,847	(1.8%)
North West	570,369	(14.1%)	61,316	(20.2%)	631,685	(14.5%)
Yorkshire & The Humber	98,964	(2.4%)	8501	(2.8%)	107,465	(2.5%)
East Midlands	128,758	(3.2%)	6676	(2.2%)	135,434	(3.1%)
West Midlands	474,262	(11.7%)	40,497	(13.3%)	514,759	(11.8%)
East of England	382,158	(9.4%)	23,294	(7.7%)	405,452	(9.3%)
London	662,375	(16.3%)	29,288	(9.6%)	691,663	(15.9%)
South East	1,190,639	(29.4%)	89,914	(29.6%)	1,280,553	(29.4%)
South West	473,188	(11.7%)	37,648	(12.4%)	510,836	(11.7%)
Townsend deprivation quintile						
1 - least deprived	733,808	(18.1%)	44,980	(14.8%)	778,788	(17.9%)
2	882,073	(21.8%)	60,389	(19.9%)	942,462	(21.6%)
3	832,270	(20.5%)	62,732	(20.6%)	895,002	(20.5%)
4	905,285	(22.3%)	77,464	(25.5%)	982,749	(22.6%)
5 - most deprived	698,229	(17.2%)	58,464	(19.2%)	756,693	(17.4%)
Whether ethnicity known						
Not Known	800,148	(19.7%)	27,009	(8.9%)	827,157	(19.0%)
Known	3,251,517	(80.3%)	277,020	(91.1%)	3,528,537	(81.0%)
Known ethnic group^a^						
Asian/British Asian	192,946	(5.9%)	8657	(3.1%)	201,603	(5.7%)
Black/Black British	96,171	(3.0%)	3397	(1.2%)	99,568	(2.8%)
Mixed	35,227	(1.1%)	1543	(0.6%)	36,770	(1.0%)
Other	81,109	(2.5%)	2634	(1.0%)	83,743	(2.4%)
White	2,846,064	(87.5%)	260,789	(94.1%)	3,106,853	(88.0%)

^a^ Percentages calculated of known ethnic group total

### Prevalence

**Fig. 2:**
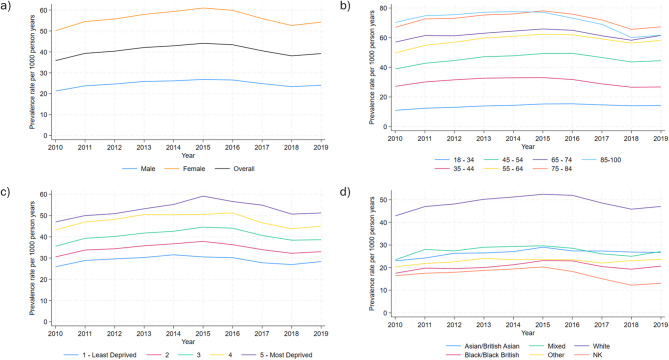
Prevalence rates of opioid and antidepressant co-prescribing per 1000 person years over time in adults aged 18–100 years, 2010–20,019, England; (**a**) overall and by sex, (**b**) by age group, (**c**) by Townsend deprivation quintile, (**d**) by ethnic group

The overall prevalence rate of co-prescribing was 40.47 per 1000 person-years (95% confidence interval (CI): 40.38–40.57). Prevalence rates increased from 35.85 per 1000 person-years in 2010 to a peak of 44.09 in 2015, and then declined over the rest of the study period to 39.20 in 2019. Figure 2 shows the co-prescribing prevalence rates in each year, overall and by sex, age group, Townsend deprivation quintile and ethnic group. Co-prescribing rates were higher in females, older age groups, people living in more deprived areas and the White ethnic group.

**Fig. 3:**
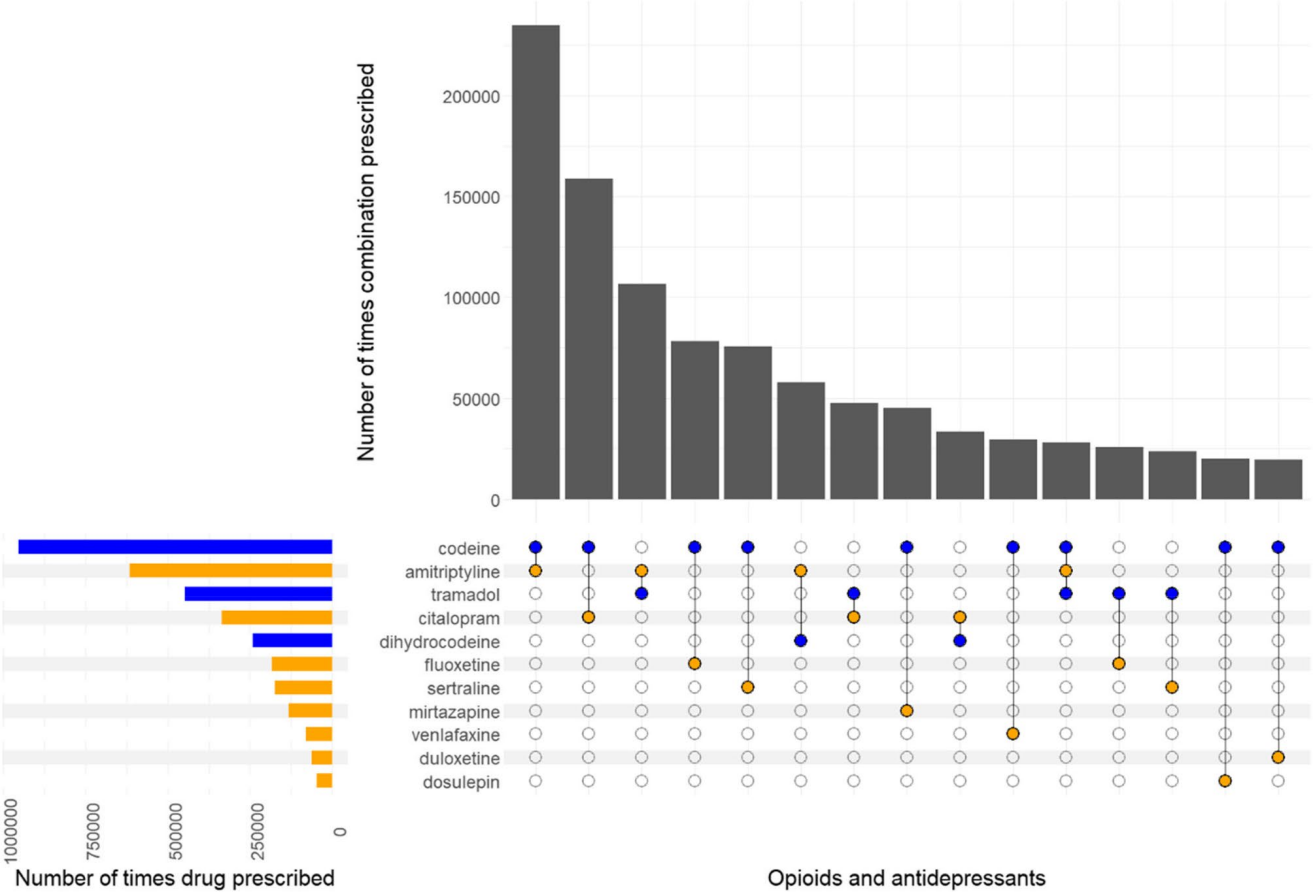
UpSet plot showing the most commonly co-prescribed opioids and antidepressants, individually and in combination

The most commonly co-prescribed opioid and antidepressant combinations are shown in Fig. 3. Codeine and amitriptyline were most commonly co-prescribed, prescribed 235,017 times in the study period (15% of all co-prescriptions) to 87,274 (29%) people. Codeine and citalopram was the second most commonly co-prescribed combination, prescribed 55,792 times (10% of co-prescriptions) to 158,812 (18%) people. The most commonly prescribed combinations were very similar when the co-prescribing period was restricted to at least 14, 28 or 365 days (Additional file 3, Figs. S3.1-S3.3).

The overall median length of the prevalent opioid and antidepressant co-prescriptions was estimated to be 29 days (IQR: 17 to 51 days). There were 23,789 (7.8%) people whose co-prescribing lasted at least 365 days. When broken down by characteristic, people in the White and older age groups had longer median lengths of co-prescription (Additional file 3, Table S3.2). The patterns of the length of the most commonly co-prescribed combinations were broadly similar, with peaks around 2 weeks and 4 weeks and then smaller numbers having longer co-prescriptions (Additional file 3, Fig. S3.4).

Contraindicated combinations of medicines were rarely co-prescribed. Of those with prevalent co-prescriptions, 42 (0.01%) people had at least one contraindicated co-prescription within the study period. Of these 15 people were co-prescribed tramadol and phenelzine, 14 were co-prescribed methadone and citalopram, and 12 were co-prescribed tramadol and tranylcypromine.

### Incidence

Overall incidence of co-prescribing was 10.60 per 1000 person-years (95% CI: 10.55–10.65). In contrast with the prevalence figures, incidence rates peaked in 2012 (11.29 per 1000 person-years). This pattern was also seen within the different groups examined (Additional file 3, Fig. S3.5). The patterns of co-prescribing mirrored those shown for prevalence; with females, older age groups, people living in more deprived areas and the White ethnic group having a higher incidence of co-prescribing.

Of the 171,226 people with incident co-prescriptions, half (86,034, 50.2%) had an antidepressant prescribed first, 54,381 (31.8%) had an opioid prescribed first, and 30,811 (18.0%) were first prescribed both on the same day. Of those with a first opioid prescription, this had been prescribed for at least 12 months before the co-prescription in 3,583 (6.6%) people, indicating long-term opioid use. The antidepressant indications studied were recorded before co-prescribing for 76,428 (44.6%) people for depression, 41,882 (24.5%) for anxiety/phobias and 39,443 (23.0%) for neuropathic pain. At least one of these indications was recorded for 110,552 (64.6%) people.

### Sensitivity analysis

The number of practices in England contributing to CPRD GOLD declined through the study period from 451 in 2010 to 105 in 2019. In the sensitivity analysis, only patients from practices that contributed for the entire study period were included. Additional file 3, Fig. S3.6 shows the overall prevalence and incidence rates over time for both populations. The original population produced slightly higher rates between 2010 and 2017 for prevalence and 2011 and 2013 for incidence, but the trends overall were very similar.

## Discussion

In this large, population-based, longitudinal study, we found that rates of co-prescribing of opioids and antidepressants are low and declining. After an initial increase from 2010 to 2015, the prevalence of co-prescribing of opioids and antidepressants decreased from 2015 to the end of the study period (2019), while the incidence decreased from 2012. Higher co-prescribing rates were shown in women, older age groups, those living in more deprived areas and people in the White ethnic group. The median length of prevalent co-prescriptions was 29 days, and the most commonly co-prescribed combination was amitriptyline and codeine. Contraindicated co-prescriptions were rare, however, fluoxetine and codeine was the fourth most commonly co-prescribed combination. Fluoxetine as a potent CYP2D6 inhibitor may reduce the effectiveness of codeine as an analgesic, as will other CYP2D6 inhibitors (sertraline, escitalopram and duloxetine) when combined with codeine or tramadol.

This is the first-time the extent of opioid and antidepressant co-prescribing in the general population of England has been described over time. Our findings do not reflect the documented increase in prescribing of opioids and antidepressants separately during this period. Jani et al. [15] found continuous increases in opioid prescribing across a similar timeframe (2006–2017) in the UK and Lalji et al. [26] found a 25% increase in antidepressant prescribing from 2015 to 2019 in England. The decline in co-prescribing shown in our study may reflect an increased awareness of the risks. The US FDA’s warning on opioid-antidepressant interactions in March 2016 [7] may have influenced the decreasing trend, as we found co-prescribing rates peaked in 2015.

Our study supports previous work showing increased prescribing in particular demographic groups. A Public Health England evidence review [14] examined co-prescribing in 5.5 million people prescribed antidepressants, opioids, gabapentinoids, benzodiazepines and Z-drugs in March 2018. Here, women had a slightly higher co-prescribing rate of any combination of these medicines than men (26% vs. 24%), as well as increasing co-prescribing rates with increasing deprivation (most deprived quintile rate 1.4 times higher than in the least deprived quintile). The review did not find a relationship with age. Previous work in the UK showed that older people, women, White ethnic groups and people living in more deprived areas were more likely to have long-term opioid use [15], reflecting similar patterns in our study. Further studies should investigate the demographic and clinical characteristics of the sub-population co-prescribed opioids and antidepressants controlling for covariates. This will enable us to further understand this difference in prescribing and how high and low risk groups contribute to this trend.

There are several strengths of this study. We used a large, population-based, representative dataset, enabling an accurate description of prevalence and incidence of co-prescribing across England over our 10-year study period for the first time. Primary care records are collected prospectively as part of patients’ routine care, meaning our study is free of any selection and recall bias. We defined opioid and antidepressant prescriptions using an exhaustive list of drug names (verified by prescribers), and antidepressant indications with an extensive list of Read codes.

A limitation of the study is that we had to estimate the end of prescription periods. Inaccurate predictions of prescription end dates directly impact our measured outcome of co-prescription as overlapping dates may be erroneous. This study also cannot account for whether prescriptions were filled, taken as prescribed or completed by the individual. Primary care data cannot capture these factors, further limiting our prescription end date calculations. While this would have a greater influence on studies examining adverse outcomes due to co-prescribing, we have confidence that these overlapping prescriptions were issued, and within a similar time frame. This study only examines prescribing within primary care, additional prescribing in secondary care would not be included. We have not separated amitriptyline prescriptions by dose, which is likely to be important as lower doses are more likely to be prescribed for pain and higher doses for depression. This may mean that a substantial amount of the co-prescribing observed with amitriptyline is all pain-related, rather than associated with mental health, and future studies may wish to make this distinction. However, there may still be adverse effects of co-prescribing opioids with amitriptyline irrespective of the indications. The study period was chosen to end before the Covid-19 pandemic, as both prescribing and data collection would have been affected by changes in how healthcare was delivered during the pandemic, and more recent data would determine whether these decreasing trends seen have continued. There was some missing data for ethnicity, even after supplementing primary care data with HES records, and a substantial proportion without recorded indications for antidepressant prescriptions.

## Conclusions

This study has provided reassuring evidence that the prevalence and incidence of opioid and antidepressant co-prescribing rates are decreasing, even as individual prescribing rates of these medicines increased. Contraindicated co-prescribing is rare, suggesting that prescribers are aware of the risks, however combinations of opioids and CYP2D6 inhibitors that may be less effective are being co-prescribed. Therefore, some education to prescribers around the use of these combinations is worth consideration. Where co-prescribing does occur, it is more common in women, older age groups, White individuals and those in more deprived areas.

Future work should investigate further the demographic and clinical characteristics of the sub-population prescribed both opioids and antidepressants, to understand whether any subgroups are at higher risk of harm. Research investigating how co-prescribing varies taking dose into account and whether there are increased risks of adverse events if co-prescribed these medicines would help prescribing practices. However, our findings that prescribing of contraindicated combinations is rare over the study period should be of reassurance to both prescribers and patients.

## Supplementary Information

Additional file 1. Code lists for CPRD GOLD used to identify antidepressants, opioids for non-cancer pain, ethnic group, neuropathic pain, depression, anxiety / phobia, cancer, terminal illness, opioid use disorder and heart failure; contraindicated combinations of opioids and antidepressants.

Additional file 2. RECORD statement. Checklist of items, extended from the STROBE statement, that should be reported in observational studies using routinely collected health data.

Additional file 3. Table S3.1. Table. Characteristics of prevalence cohort by co-prescription length, aged 18-100 years, England 2010–2019. Table S3.2. Median and interquartile range (IQR) of co-prescription length in days, overall and by characteristics for all co-prescriptions and those lasting at least 90 days. Fig. S3.1. UpSet plot showing the most commonly co-prescribed opioids and antidepressants with at least 14 days overlap, individually and in combination. Fig. S3.2. UpSet plot showing the most commonly co-prescribed opioids and antidepressants with at least 28 days overlap, individually and in combination. Fig. S3.3. UpSet plot showing the most commonly co-prescribed opioids and antidepressants with at least 365 days overlap, individually and in combination. Fig. S3.4. Length of prevalent co-prescribing of the most common medicine combinations, in days, up to 365+ days. Fig. S3.5. Incidence rates of opioid and antidepressant co-prescribing per 1000 person years over time in adults aged 18-100 years, 2010-20019, England; a) overall and by sex, b) by age group, c) by Townsend deprivation quintile, d) by ethnic group. Fig. S3.6. Comparison of original and sensitivity populations over time for a) prevalence and b) incidence of opioid and antidepressant co-prescribing.

## Data Availability

The data that support the findings of this study are not publicly available. They were provided by the Clinical Practice Research Datalink (CPRD) under license and according to CPRD’s data governance process. Researchers wishing to access the data must apply to CPRD, and requests are subject to protocol approval (https://cprd.com/data-access). Queries about the study data and results should be directed to the corresponding author (RHJ). Code lists are available in the additional files and via the HDR UK Phenotype Library.

## Abbreviations

ARC EM: Applied Research Collaboration East Midlands
CI: Confidence interval
CPRD: Clinical Practice Research Datalink
HES: Hospital episode statistics
IQR: Interquartile range
NICE: National Institute for Health and Care Excellence
NIHR: National Institute for Health and Care Research
PPIE: Patient and Public Involvement and Engagement
RECORD: REporting of studies Conducted using Observational Routinely collected health Data
RDG: Research Data Governance
US FDA: United States Food and Drug Administration
