# Supplementary material for: Co-prescribing of antidepressants and opioids for non-cancer pain in England, 2010–2019: a descriptive study using CPRD primary care electronic health records
Source: BMC Prim Care. 2025 Aug 16;26:254. doi: 10.1186/s12875-025-02956-1 (PMC12357414; doi:10.1186/s12875-025-02956-1)
Supplement: Supplementary file 3 — Additional file 3. Table S3.1. Table. Characteristics of prevalence cohort by co-prescription length, aged 18-100 years, England 2010–2019. Table S3.2. Median and interquartile range (IQR) of co-prescription length in days, overall and by characteristics for all co-prescriptions and those lasting at least 90 days. Fig. S3.1. UpSet plot showing the most commonly co-prescribed opioids and antidepressants with at least 14 days overlap, individually and in combination. Fig. S3.2. UpSet plot showing the most commonly co-prescribed opioids and antidepressants with at least 28 days overlap, individually and in combination. Fig. S3.3. UpSet plot showing the most commonly co-prescribed opioids and antidepressants with at least 365 days overlap, individually and in combination. Fig. S3.4. Length of prevalent co-prescribing of the most common medicine combinations, in days, up to 365+ days. Fig. S3.5. Incidence rates of opioid and antidepressant co-prescribing per 1000 person years over time in adults aged 18-100 years, 2010-20019, England; a) overall and by sex, b) by age group, c) by Townsend deprivation quintile, d) by ethnic group. Fig. S3.6. Comparison of original and sensitivity populations over time for a) prevalence and b) incidence of opioid and antidepressant co-prescribing. [file 12875_2025_2956_MOESM3_ESM.docx]

Additional File 3

Additional results

Contents

[Additional Table S3.1. Characteristics of prevalence cohort by co-prescription length, aged 18-100 years, England 2010–2019 2](#_Toc177379989)

[Additional Table S3.2. Median and interquartile range (IQR) of co-prescription length in days, overall and by characteristics for all co-prescriptions and those lasting at least 90 days 3](#_Toc177379990)

[Additional Fig. S3.1. UpSet plot showing the most commonly co-prescribed opioids and antidepressants with at least 14 days overlap, individually and in combination 4](#_Toc177379991)

[Additional Fig. S3.2. UpSet plot showing the most commonly co-prescribed opioids and antidepressants with at least 28 days overlap, individually and in combination 5](#_Toc177379992)

[Additional Fig. S3.3. UpSet plot showing the most commonly co-prescribed opioids and antidepressants with at least 365 days overlap, individually and in combination 6](#_Toc177379993)

[Additional Fig. S3.4. Length of prevalent co-prescribing of the most common medicine combinations, in days, up to 365+ days 7](#_Toc177379994)

[Additional Fig. S3.5. Incidence rates of opioid and antidepressant co-prescribing per 1000 person years over time in adults aged 18-100 years, 2010-20019, England; a) overall and by sex, b) by age group, c) by Townsend deprivation quintile, d) by ethnic group. 8](#_Toc177379995)

[Additional Fig. S3.6. Comparison of original and sensitivity populations over time for a) prevalence and b) incidence of opioid and antidepressant co-prescribing. 9](#_Toc177379996)

# Additional Table S3.1. Characteristics of prevalence cohort by co-prescription length, aged 18-100 years, England 2010–2019

|  | Co-prescription length | | | |
| --- | --- | --- | --- | --- |
|  | ≥90 days | | ≥365 days | |
|  |  |  |  |  |
| Overall | 61714 | (100.0%) | 26543 | (100.0%) |
|  |  |  |  |  |
| Age category |  |  |  |  |
| 18-34 | 5796 | (9.4%) | 1463 | (5.5%) |
| 35-44 | 9435 | (15.3%) | 3773 | (14.2%) |
| 45-54 | 12966 | (21.0%) | 6125 | (23.1%) |
| 55-64 | 13050 | (21.1%) | 6496 | (24.5%) |
| 65-74 | 10316 | (16.7%) | 4706 | (17.7%) |
| 75-84 | 7166 | (11.6%) | 2921 | (11.0%) |
| 85-100 | 2985 | (4.8%) | 1059 | (4.0%) |
|  |  |  |  |  |
| Sex |  |  |  |  |
| Male | 18655 | (30.2%) | 8405 | (31.7%) |
| Female | 43059 | (69.8%) | 18138 | (68.3%) |
|  |  |  |  |  |
| Region |  |  |  |  |
| North East | 1556 | (2.5%) | 868 | (3.3%) |
| North West | 13467 | (21.8%) | 7293 | (27.5%) |
| Yorkshire & The Humber | 1771 | (2.9%) | 670 | (2.5%) |
| East Midlands | 1602 | (2.6%) | 506 | (1.9%) |
| West Midlands | 8328 | (13.5%) | 3841 | (14.5%) |
| East of England | 4690 | (7.6%) | 1471 | (5.5%) |
| London | 5275 | (8.5%) | 1857 | (7.0%) |
| South East | 17328 | (28.1%) | 6996 | (26.4%) |
| South West | 7697 | (12.5%) | 3041 | (11.5%) |
|  |  |  |  |  |
| Townsend deprivation quintile |  |  |  |  |
| 1 - least deprived | 8546 | (13.8%) | 3196 | (12.0%) |
| 2 | 11874 | (19.2%) | 4672 | (17.6%) |
| 3 | 12669 | (20.5%) | 5413 | (20.4%) |
| 4 | 16121 | (26.1%) | 7319 | (27.6%) |
| 5 - most deprived | 12504 | (20.3%) | 5943 | (22.4%) |
|  |  |  |  |  |
| Ethnicity known |  |  |  |  |
| Not Known | 5115 | (8.3%) | 2126 | (8.0%) |
| Known | 56599 | (91.7%) | 24417 | (92.0%) |
|  |  |  |  |  |
| Known ethnic group^a^ |  |  |  |  |
| Asian/British Asian | 1302 | (2.3%) | 397 | (1.6%) |
| Black/Black British | 508 | (0.9%) | 121 | (0.5%) |
| Mixed | 242 | (0.4%) | 79 | (0.3%) |
| Other | 472 | (0.8%) | 169 | (0.7%) |
| White | 54075 | (95.5%) | 23651 | (96.9%) |

^a^ Percentages calculated of known ethnic group total

# Additional Table S3.2. Median and interquartile range (IQR) of co-prescription length in days, overall and by characteristics for all co-prescriptions and those lasting at least 90 days

|  | Overall | | 90+ days overlap | |
| --- | --- | --- | --- | --- |
|  | Median | (IQR) | Median | (IQR) |
| Overall | 29 | (17-51) | 166 | (117-274) |
| Sex |  |  |  |  |
| Male | 29 | (17-53) | 169 | (118-285) |
| Female | 28 | (16-51) | 164 | (117-270) |
| Age category at study entry (years) |  |  |  |  |
| 18-34 | 22 | (13-32) | 152 | (113-240) |
| 35-44 | 26 | (15-43) | 162 | (116-265) |
| 45-54 | 28 | (16-48) | 165 | (117-276) |
| 55-64 | 29 | (17-56) | 169 | (118-283) |
| 65-74 | 29 | (17-57) | 167 | (117-279) |
| 75-84 | 29 | (17-57) | 167 | (118-272) |
| 85-100 | 29 | (17-61) | 169 | (117-275) |
| Region |  |  |  |  |
| North East | 29 | (15-57) | 176 | (120-308) |
| North West | 29 | (17-57) | 171 | (119-294) |
| Yorkshire & The Humber | 26 | (14-43) | 167 | (117-278) |
| East Midlands | 29 | (17-57) | 162 | (117-262) |
| West Midlands | 29 | (17-55) | 168 | (118-278) |
| East of England | 29 | (16-50) | 160 | (116-253) |
| London | 24 | (15-41) | 159 | (115-259) |
| South East | 28 | (16-49) | 163 | (116-266) |
| South West | 29 | (17-52) | 164 | (117-268) |
| Townsend deprivation quintile |  |  |  |  |
| 1 - least deprived | 29 | (17-53) | 162 | (116-261) |
| 2 | 29 | (17-52) | 164 | (117-267) |
| 3 | 29 | (16-51) | 165 | (117-274) |
| 4 | 29 | (16-52) | 167 | (117-278) |
| 5 - most deprived | 28 | (16-49) | 169 | (118-285) |
| Ethnic group |  |  |  |  |
| Asian/British Asian | 25 | (15-38) | 158 | (115-249) |
| Black/Black British | 23 | (15-37) | 150 | (113-236) |
| Mixed | 24 | (14-40) | 156 | (116-241) |
| Other | 26 | (15-41) | 157 | (114-262) |
| White | 29 | (17-53) | 166 | (117-276) |
| Not known | 29 | (17-51) | 166 | (117-273) |
| Year of prescription |  |  |  |  |
| 2010 | 29 | (17-58) | 180 | (121-322) |
| 2011 | 28 | (15-49) | 164 | (117-272) |
| 2012 | 28 | (16-49) | 164 | (117-274) |
| 2013 | 28 | (17-50) | 163 | (116-268) |
| 2014 | 28 | (17-52) | 165 | (117-271) |
| 2015 | 28 | (17-51) | 158 | (115-248) |
| 2016 | 28 | (17-50) | 162 | (116-261) |
| 2017 | 28 | (17-49) | 166 | (116-274) |
| 2018 | 29 | (17-51) | 169 | (118-287) |
| 2019 | 26 | (16-43) | 140 | (110-199) |

# Additional Fig. S3.1. UpSet plot showing the most commonly co-prescribed opioids and antidepressants with at least 14 days overlap, individually and in combination


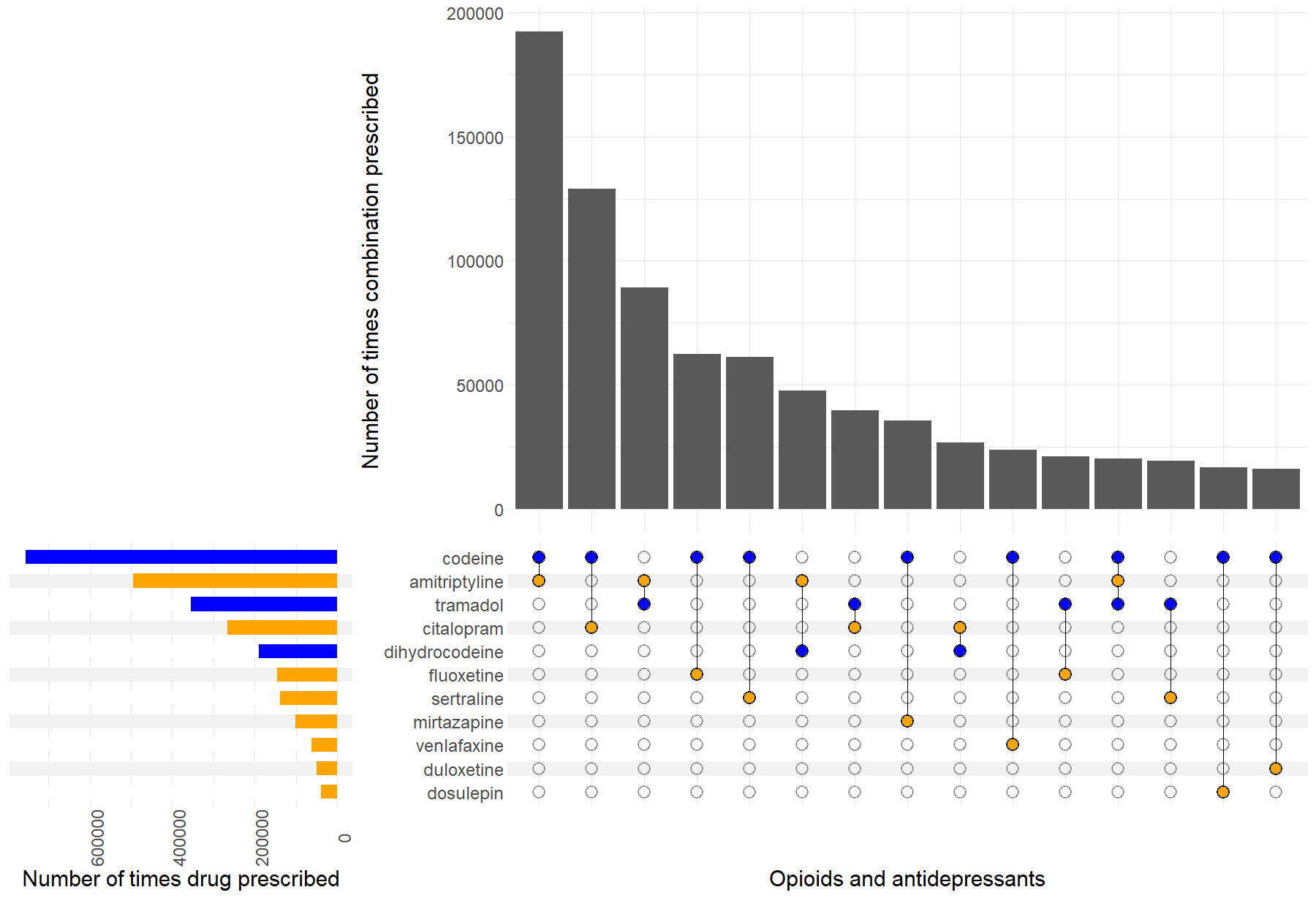


# Additional Fig. S3.2. UpSet plot showing the most commonly co-prescribed opioids and antidepressants with at least 28 days overlap, individually and in combination


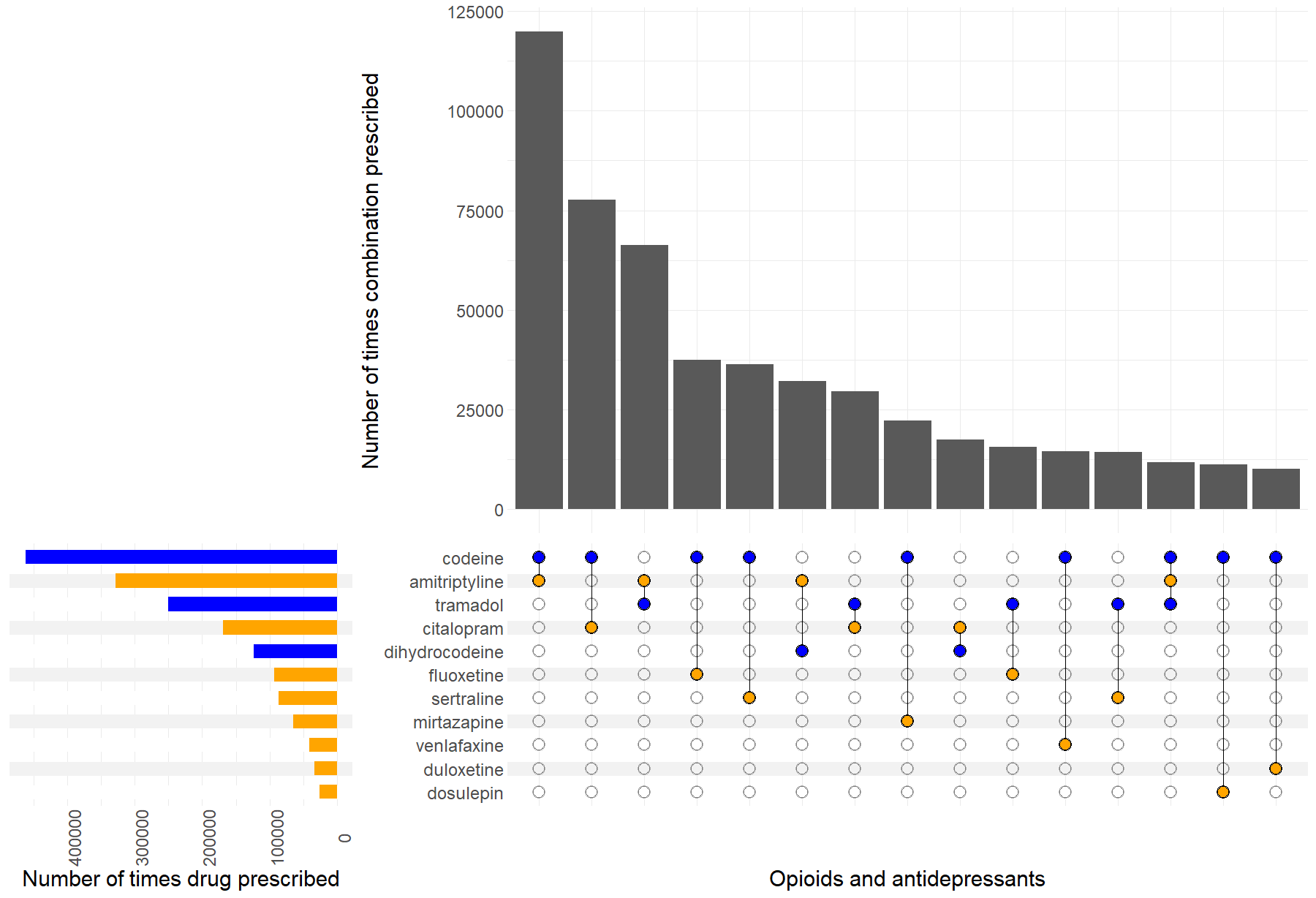


# Additional Fig. S3.3. UpSet plot showing the most commonly co-prescribed opioids and antidepressants with at least 365 days overlap, individually and in combination


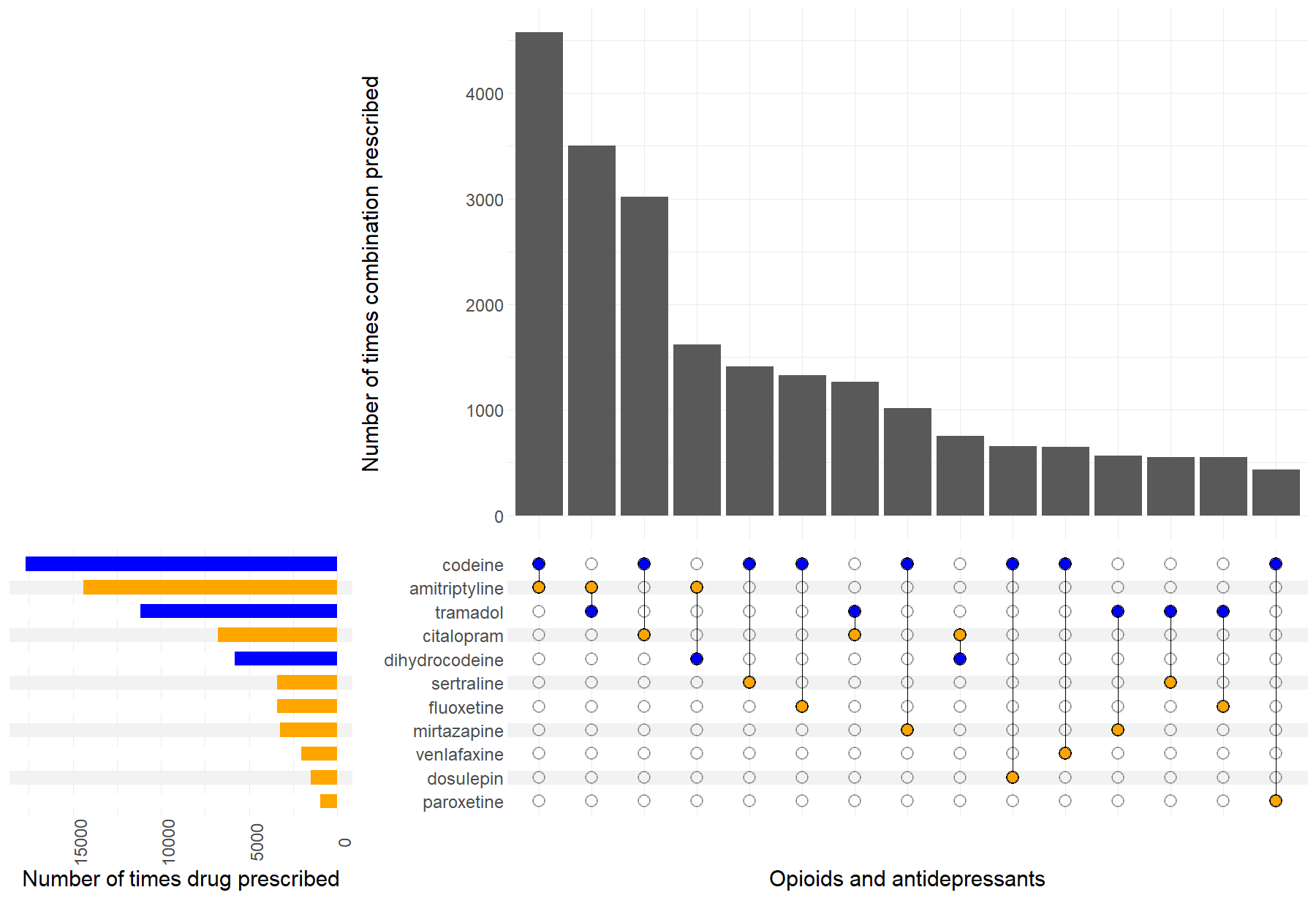


# Additional Fig. S3.4. Length of prevalent co-prescribing of the most common medicine combinations, in days, up to 365+ days

# Additional Fig. S3.5. Incidence rates of opioid and antidepressant co-prescribing per 1000 person years over time in adults aged 18-100 years, 2010-20019, England; a) overall and by sex, b) by age group, c) by Townsend deprivation quintile, d) by ethnic group.

| a) | 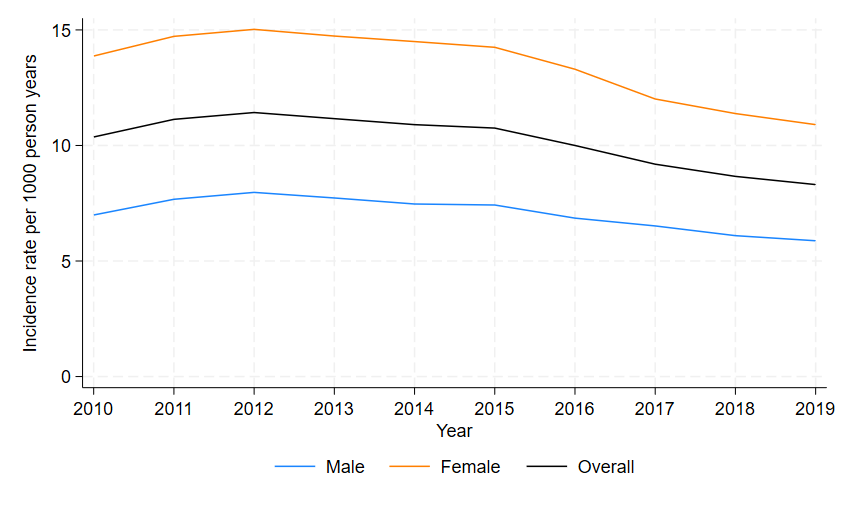 | b) | 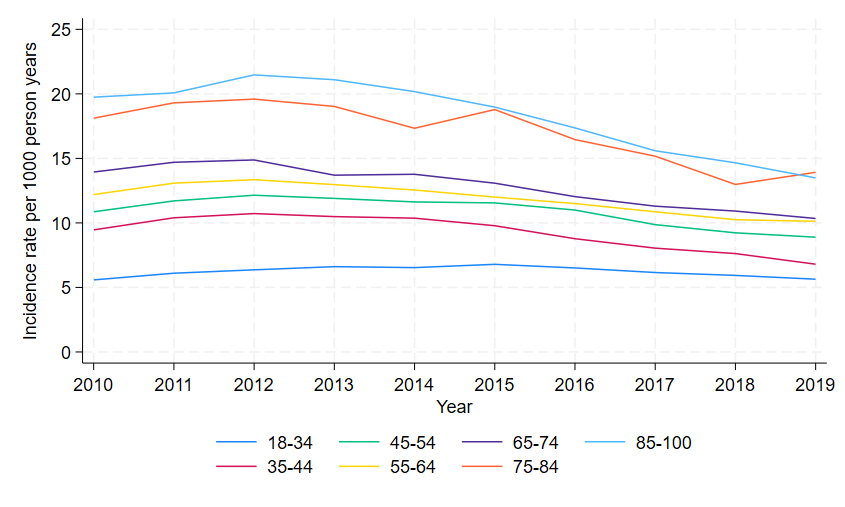 |
| --- | --- | --- | --- |
| c) | 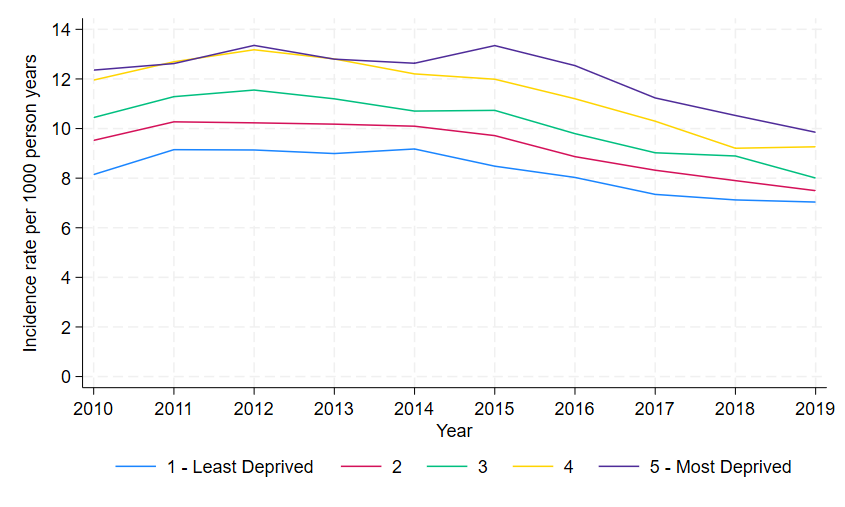 | d) | 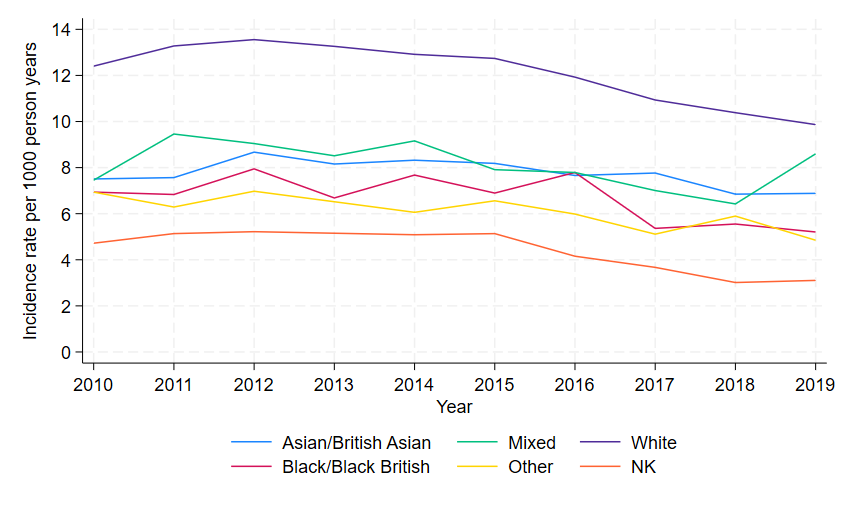 |

# Additional Fig. S3.6. Comparison of original and sensitivity populations over time for a) prevalence and b) incidence of opioid and antidepressant co-prescribing.

| a) | 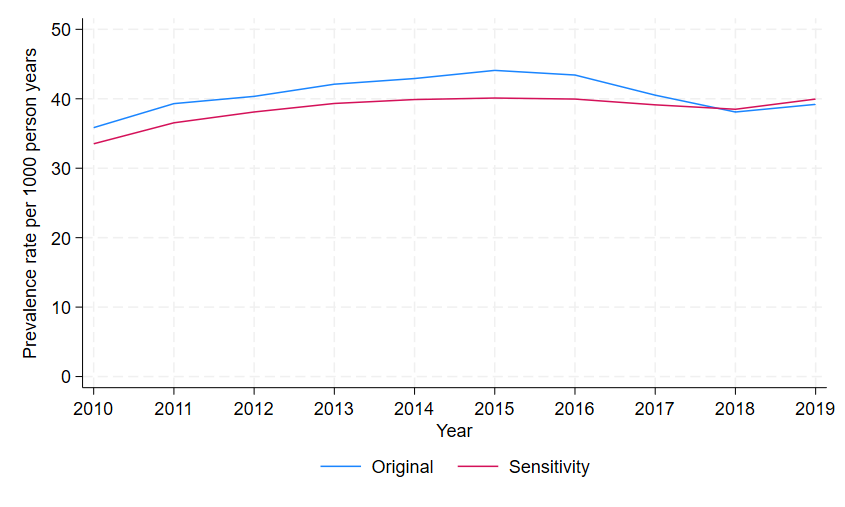 |
| --- | --- |
| b) | 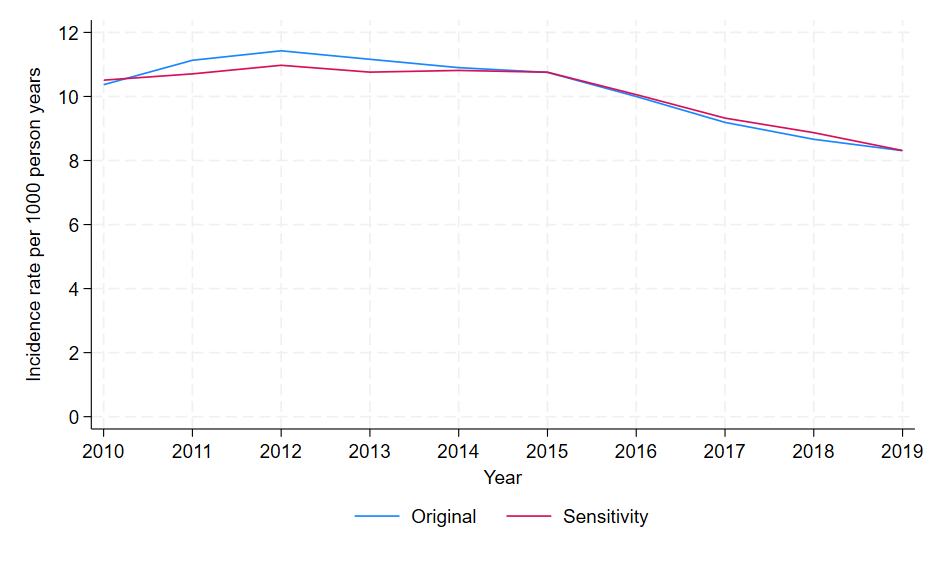 |
